# Optically Triggered Control of the Charge Carrier Density in Chemically Functionalized Graphene Field Effect Transistors

**DOI:** 10.1002/chem.202000431

**Published:** 2020-03-27

**Authors:** Zian Tang, Antony George, Andreas Winter, David Kaiser, Christof Neumann, Thomas Weimann, Andrey Turchanin

**Affiliations:** ^1^ Institute of Physical Chemistry Friedrich Schiller University Jena Lessingstraße 10 07743 Jena Germany; ^2^ Physikalisch-Technische Bundesanstalt (PTB) Bundesallee 100 38116 Braunschweig Germany; ^3^ Jena Center for Soft Matter Philosophenweg 7 07743 Jena Germany

**Keywords:** 2D materials, chemical functionalization, field effect transistors, graphene, photoresponsive devices

## Abstract

Field effect transistors (FETs) based on 2D materials are of great interest for applications in ultrathin electronic and sensing devices. Here we demonstrate the possibility to add optical switchability to graphene FETs (GFET) by functionalizing the graphene channel with optically switchable azobenzene molecules. The azobenzene molecules were incorporated to the GFET channel by building a van der Waals heterostructure with a carbon nanomembrane (CNM), which is used as a molecular interposer to attach the azobenzene molecules. Under exposure with 365 nm and 455 nm light, azobenzene molecules transition between *cis* and *trans* molecular conformations, respectively, resulting in a switching of the molecular dipole moment. Thus, the effective electric field acting on the GFET channel is tuned by optical stimulation and the carrier density is modulated.

## Graphical abstract

**Optically switchable graphene FET**: Via van der Waals assembly of an approximately 1 nm thick azobenzene functionalized molecular nanosheets with single layer graphene, optically responsive field‐effect transistors are demonstrated. The electrical carrier density of these devices can be modulated using optical stimuli, 365 nm and 455 nm light.

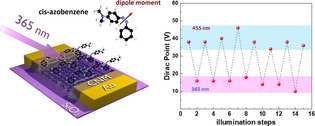

Graphene field effect transistors (GFETs) have attracted immense research attention due to their superior physical properties including high carrier mobility, ambipolar transport behaviour, high thermal conductivity, flexibility, transparency and high mechanical/chemical stability.^1^ The development of chemical vapour deposition (CVD) technique to synthesise large area high electronic quality graphene^2^ accelerated the evolution of GFETs and identified them as a promising candidate for ultrathin flexible electronics and sensing.^3^ Controlling the electronic properties of GFET channel is highly desirable for the design and realization of devices and related applications. Charge carrier doping is an effective method to control the electronic properties of graphene. In recent years, several approaches were developed which enable effective control of charge carriers in GFET channels, including covalent functionalization of graphene with functional molecules,^4^ substitutional doping of other elements replacing carbon,^5^ and noncovalent functionalization by physisorption4b, 6 or by interaction with self‐assembled monolayers.5d, 7 However, these methodologies possess certain drawbacks. The covalent functionalization of graphene requires the generation of dangling bonds4b by chemical or plasma treatment resulting in increased charge carrier scattering leading to the decrease of conductivity and carrier mobility.4b Substitutional doping of graphene with other elements results in similar issues.^8^ The noncovalent functionalization is a promising way towards non‐destructive functionalization of graphene FETs, however, simple physisorption of functional molecules results in unstable doping of graphene, the doping effect varies significantly due to desorption/resorption of the adsorbates at different temperatures and humidity.^9^

The functionalization of the graphene channel with responsive molecules provides additional opportunities to incorporate novel functionalities into GFETs. Such molecules can be used to modulate charge carrier concentration in the graphene channel.^10^ However, the chemically inert nature of graphene restricts the direct covalent functionalization of graphene surfaces.4b, 11 In 2014, Woszczyna et al. demonstrated an efficient non‐destructive chemical functionalization method using atomically thin amino terminated carbon nanomembranes (NH_2_‐CNM)^12^ as a molecular ∼1 nm thick interposer.^13^ In this approach, a van der Waals (vdW) heterostructure is fabricated with graphene and NH_2_‐CNM. As the amino groups of the CNM can be covalently functionalized, this method enables non‐destructive functionalization of graphene while its original structural/electronic quality is preserved.^13^

In this contribution, we demonstrate the integration of optically active azobenzene molecules with graphene channel to realize optically switchable GFET devices. The azobenzene molecules undergo reversible transformations between *cis* and *trans* molecular conformations under exposure to 365 nm and 455 nm light, respectively.1c, 10a The azobenzene molecule in its *cis*‐conformation has a dipole moment of ≈3 D while in its *trans*‐conformation does not possess any dipole moment.6b, 14 By switching the azobenzene molecules between *cis*‐ (by 365 nm light) and *trans*‐ (by 455 nm light) conformation, the effective molecular field acting on the GFET channel can be modulated, thus changing doping level of the device.10a, 15 In order to incorporate the graphene channel with azobenzene molecules, we prepared a van der Walls (vdW) heterostructure of azobenzene functionalized CNM (azo‐CNM) with graphene. This is achieved by transferring a layer of the functionalized NH_2_‐CNM on top of an array of GFET devices. The optically switchable hybrid azo‐GFET device in its two different conformations is schematically illustrated in Figure 1. The successful functionalization was confirmed by a detailed X‐ray photoelectron spectroscopy (XPS) analysis. Electric transport measurements after irradiation with 365 nm and 455 nm light were performed to understand the operational characteristics of the optically switchable azo‐GFET devices. Based on these data, we correlate the density of the functional azobenzene groups on the azo‐GFET channel with the transport characteristics and therewith rationalize the observed switching behaviour.

**Figure 1:**
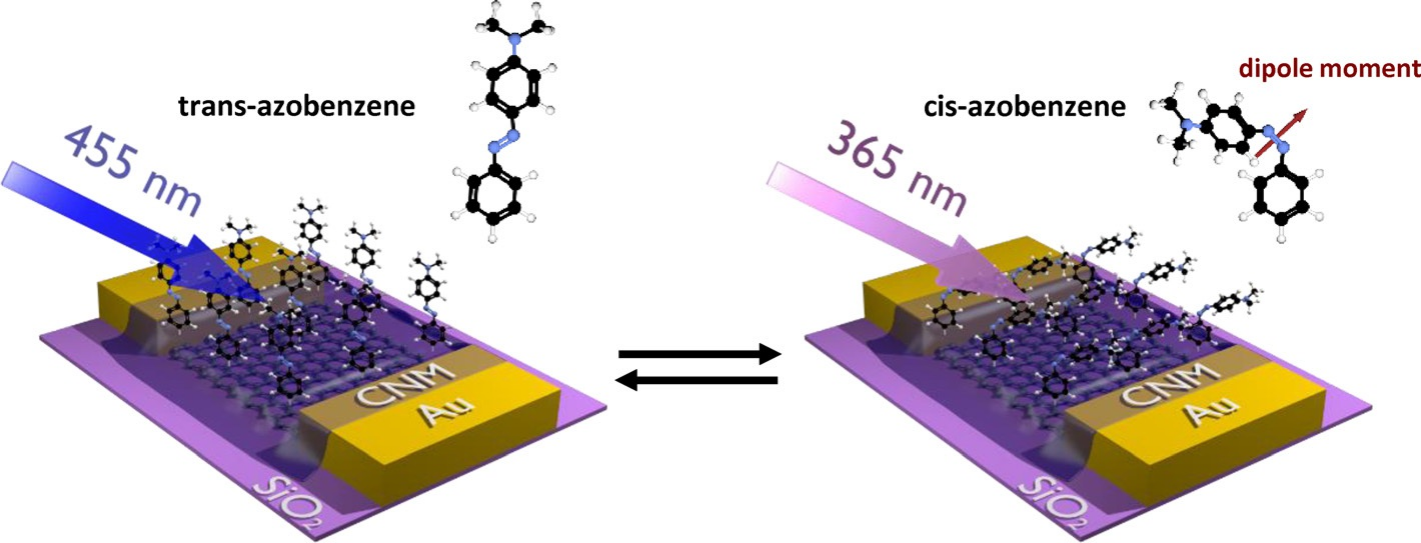
Schematic illustration of the working principle of an optically switchable GFET device. The azobenzene molecules immobilized on the amino‐CNM undergo *cis*‐ and *trans*‐transformations by exposure with 365 nm and 455 nm light, respectively. The red arrow in the magnified azobenzene molecule points towards the direction of the molecular dipole moment.

First, we describe the synthesis of azo‐CNMs. To this end, the NH_2_‐CNMs were prepared by electron beam induced crosslinking of 4'‐nitro‐1,1'‐biphenyl‐4‐thiol (NBPT) self‐assembled monolayers (SAMs) on gold/mica substrates.^13^, ^16^ Subsequently, their functionalization with azobenzene was performed using the NHS‐ester coupling reaction, as schematically shown in the supporting information (SI), Figure S1. The efficiency of the functionalization was studied by XPS. Thus, based on the attenuation of the substrate Au 4f signal (see Figure S2), an increase of the NH_2_‐CNM effective thickness from 1.2 nm to 1.4 nm was detected after the functionalization. In Figure 2 a the XP N 1 s spectra of a NH_2_‐CNM before and after the functionalization with azobenzene are presented. In the spectrum of a pristine NH_2_‐CNM (top) the peak at 399.4 eV (blue) corresponds to amino (‐NH_2_) groups. After the functionalization, the N 1 s spectrum consists of two peaks showing in comparison to the pristine NH_2_‐CNM more than one type of nitrogen. The peak at 399.1 eV (blue) is due to both amino and tertiary amine^17^ groups; the peak at 400.3 eV (red) corresponds to nitrogen in the azo groups^18^ and in the amide^19^ groups connecting the azobenzene molecules to the CNM. Assuming the *trans*‐conformation for the azobenzene and analysing quantitatively the N 1 s intensities before and after the functionalization, we estimate that 20(±3) % of the amino groups initially present in an NH_2_‐CNM are functionalized with azobenzene (see SI for details). In Figure 2 b the respective XP C 1 s signals are shown. After the functionalization, we observe an enhancement of the intensity at 285.5 eV (blue), which is due to carbon connected to azo and tertiary amine groups in the azobenzene. The amide group that links azobenzene to the CNM results in an increase (≈20 %) of the C1s intensity at 288.1 eV (green) corresponding to a highly oxidized carbon species.^20^ Note that due to an attenuation caused by the grafted azobenzene layer, the C 1 s peaks representing the aromatic (284.3 eV, red) and ketone (286.9 eV, orange) carbon species became less intensive. In comparison to the pristine NH_2_‐CNM sample, we observe also an attenuation of the S 2p signal in the functionalized sample, Figure S2. All these findings clearly demonstrate a successful grafting of the azobenzene molecules to the terminal amino groups of NH_2_‐CNMs and therewith the synthesis of azo‐CNMs.

**Figure 2:**
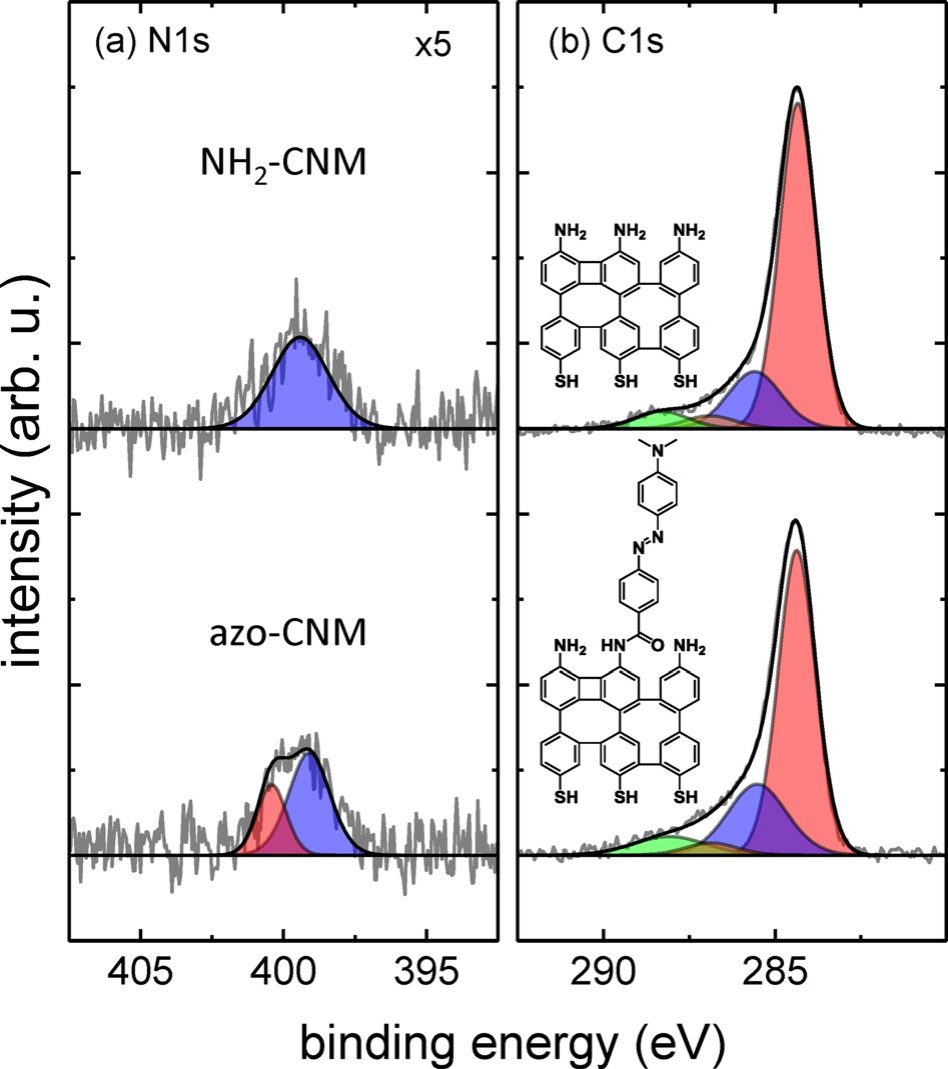
High‐resolution N1s (a) and C1s (b) XP spectra of NH_2_‐CNM and azo‐CNM.

Next, we describe the fabrication and characterization of azo‐CNM/GFET devices. The single layer graphene (SLG) was grown by chemical vapour deposition (CVD) on Cu foils and transferred onto a Si substrate with 600 nm thermally grown SiO_2_ layer using the non‐destructive transfer method.^21^ The graphene quality was controlled by optical microscopy and Raman spectroscopy (Figure S3). The GFET devices were fabricated by defining an array of source‐drain contacts (30 nm Au/ 5 nm Ti) on the previously transferred graphene by electron beam lithography, metallization, lift‐off and etching procedure.^13^ After the basic optical and electrical characterization of the device arrays (SI, Figure S4 a‐c), an azo‐CNM was transferred on top of the devices. In this way, the vdW heterostructure was formed at the channel region of the GFET device as shown schematically in Figure 1. The typical field‐effect mobility of the charge carriers in the fabricated devices was ≈2500 cm/Vs.

To investigate the electrical characteristics and to test the optical switchability of the heterostructure devices, we have performed a series of field‐effect transport measurements with and without optical excitation. We used two light emitting diodes (LED) with emission wavelengths of 365 nm and 455 nm to optically excite the azobenzene molecules to *cis* and *trans* conformation. We initially measured the pristine GFET devices before transferring the azo‐CNM to test any effect on pristine devices within the range of illumination intensities used for the switching experiments. Transfer curves of a pristine GFET device before and after illumination with 365 nm and 455 nm wavelengths are presented in SI Figure S4a and Figure S4b, respectively. Each transfer curve was recorded under dark condition, immediately after 15 minutes of illumination. The results show no significant difference in the transfer curves and confirm that the light has no noticeable effect on pristine graphene devices within the range of illumination intensities we used. We have also tested a GFET device with a transferred NH_2_‐CNM (without azobenzene functionalization) on top with similar illumination conditions as presented in SI Figures S4 d and e, which also did not show any significant difference in the transfer characteristics after irradiation.

We present in Figure 3 a the typical transfer characteristics of an azo‐CNM/GFET device, immediately after irradiation with 365 or 455 nm for about 1 hour. The measurements were recorded in dark after the light exposure. By exposing the device with the two different wavelengths, the charge neutrality point (commonly known as Dirac point) of the device can be switched reversibly. When the device is exposed with the light of 365 nm wavelength, the Dirac point shifts to lower values of the gate voltage, V_G_, which shows a doping of the device with negative charge carriers. When the device is exposed with the light of 455 nm wavelength, the Dirac point shifts towards a higher gate voltage, indicating removal of the *n*‐doping effect caused by the exposure of shorter wavelength light. In Figure 3 b, we present the time‐dependent switching behaviour of the same azo‐CNM/GFET device for two exposure cycles with 365 nm and 455 nm. The transfer curves were recorded every minute during exposure until the transfer characteristics did not any longer shift significantly. Before exposure with 365 nm wavelength, the Dirac point of the device was at 131 V. After exposure to 365 nm wavelength for ≈1 h the Dirac point shifted to 94 V, corresponding to a doping with negative charge carriers. When the device is exposed with 455 nm wavelength the induced *n*‐doping was lifted and the Dirac point of the device shifted back to 125 V. The corresponding transfer curves for each time dependent exposure are presented in Figure S5. As was demonstrated earlier,^22^ the photoswitching of azobenzene molecules incorporated into alkanethiol SAMs follows a simple exponential behaviour. Therefore, the shift of the Dirac point was fitted with single exponential functions in order to obtain the respective time constants (see Figure S5e). Both *trans* to *cis* and *cis* to *trans* photoswitching were found to be of a similar value of 10(±0.5) min. Note that in our experiments the intensity of the 455 nm light was a factor of 1.5 higher than the intensity of the 365 nm light (see Experimental for details), which implies that the *trans* to *cis* switching is more efficient than the *cis* to *trans*. These results are in a good qualitative agreement with the respective photoswitching cross sections measured for different azobenzene SAMs on gold.^23^ Employing this finding, we used subsequent 15 min irradiations with 365 nm and 455 nm to modulate a reversible shift of the Dirac point by ∼20 V in the azo‐CNM/GFETs (see Figure S6).

**Figure 3:**
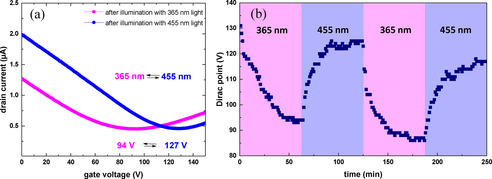
Photoresponsive behaviour of azo‐CNM/GFET devices. (a) The transfer curves of an azo‐CNM/GFET after illumination for 60 min with 365 nm and 455 nm light. (b) Time dependent change of the position of Dirac point until sequential illumination with 365 nm and 455 nm light; the corresponding curves are presented in Figure S5.

In the following, we analyse the observed photoswitching behaviour considering the surface density azobenzene molecules on the azo‐CNM/GFET channel and the respective induced electrical field in their *cis* conformation. In Figure 4 a, we show the transport curves of a device before and after a single irradiation with 365 nm wavelength. The black curve represents the transfer characteristics of the pristine device. The Dirac point of the device is at 69 V. The purple curve represents the transfer characteristics after irradiation with 365 nm wavelength, which results is a shift of the Dirac point to 35 V. The grey curve represents the transfer curve of the device after thermal relaxation at room temperature (RT) for 48 hours, which demonstrates that the device restored the original position of the Dirac point, that is, its doping level. We estimate from the time dependent measurements the time constant of the thermally induced *cis* to *trans* relaxation to ≈15 h, which is in good qualitative agreement with an earlier study for azobenzene SAMs.^24^ We attribute the observed photoswitching behaviour on the modulation of the Dirac point, that is, doping level, of the azo‐GFET devices to the variation of the local electric field acting on the graphene channel due to the *cis* and *trans* conformations of the azobenzene molecules triggered by the light excitation. Assuming that initially all molecules are in the thermally stable *trans*‐state with a dipole moment$\;\;\mu_{trans}\approx 0\;D$ , no effective electric field is present in the azobenzene layer before irradiation with 365 nm light. After the irradiation, the azobenzene molecules adopt the *cis*‐conformation with a dipole moment of 3D .^25^ The effective electric field induced by the molecules can be calculated then (see Figure 4 b) using the relation [Eq. (1)]:^26^

(1) $$E=N\left(\frac{\sin\theta \mu_{mol}}{\epsilon_{r}\epsilon_{0}d_{mol}}\right)$$

**Figure 4:**
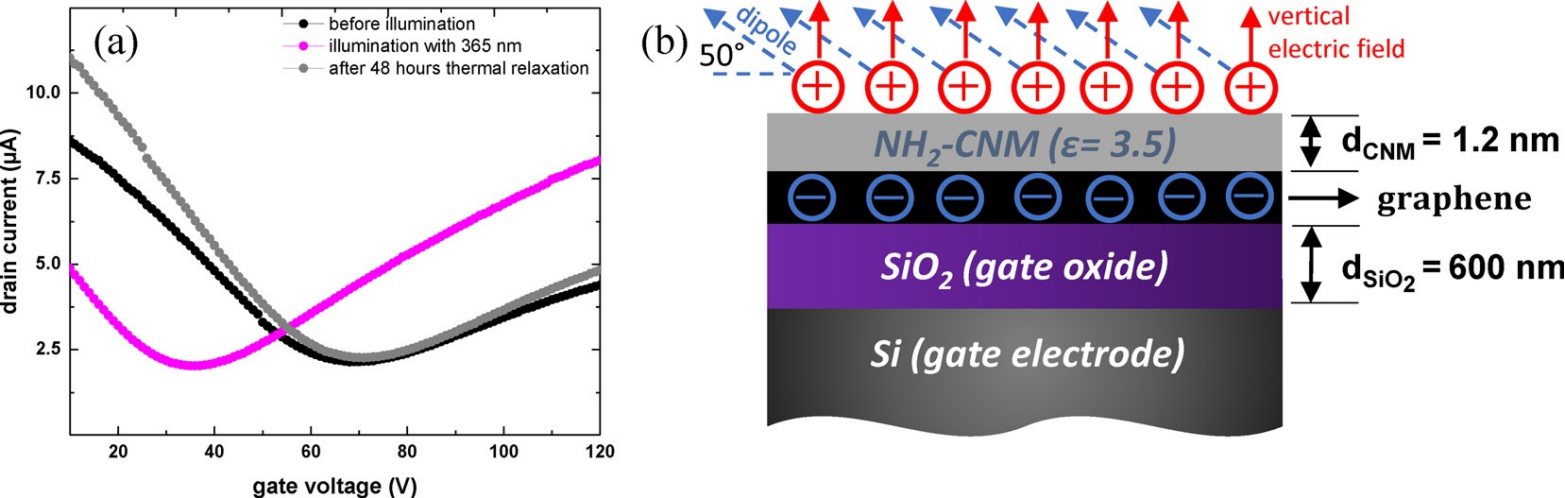
(a) Transfer curves of an azo‐CNM/GFET device recorded before exposure, after exposure with 365 nm light and after thermal relaxation at RT for two days. (b) Schematic illustration of the induced electric field effect due to the conformational change of azobenzene from *trans* to *cis*.

where *N* and *d_mol_* are the surface density and length of the azobenzene molecules in the *cis*‐conformation, *μ_mol_* is the dipole moment of the molecule, *θ* is the angle between the dipole moment and the surface, which is approximately 50°25a and *ϵ_r_* is the relative dielectric constant within the azobenzene layer.^26^ Based on the surface density of the amino groups in a NH_2_‐CNM and their functionalization efficiency with the azobenzene molecules obtained by XPS, we can estimate the surface density of the azobenzene species on the channel of an azo‐CNM/GFET. The density of amino groups in a NH_2_‐CNM is ∼3.3 molecules/nm^2^ (i.e., 3.3×10^14^ cm^−2^).^12^ Considering that only ∼20 % of these groups are functionalized with azobenzene, their surface density is then 6.6×10^13^/ cm^2^. The length of the azobenzene molecule in the *cis*‐conformation is ≈0.7 nm. The typical relative dielectric constant of organic molecules can be taken as ≈3.^26^, ^27^ Therewith, the vertical effective electric field within the azobenzene layer is calculated to be 272 MV m^−1^. With the azobenzene thickness in its *cis*‐conformation of 0.7 nm, the effective gate voltage applied to the NH_2_‐CNM, acting as a gate dielectric for the graphene channel, is 0.19 V. Figure 4 b schematically illustrates this consideration. Thus, the variation of the charge carrier concentration induced by the presence of the molecular dipoles can be calculated using Equation (2):

(2) $${\Delta n}_{cis\to trans}=\frac{C_{CNM}{\Delta V}_{cis\to trans}}{e}$$

where *C_CNM_* is the capacitance of NH_2_‐CNM, ${\Delta V}_{cis\to trans}$ is the variation in the top gate voltage caused by the molecular dipoles and *e* is the charge of an electron. The capacitance of NH_2_‐CNM is calculated as 2.6×10^−2^ F m^−2^ using Equation (3):

(3) $$C_{CNM}=\;\epsilon_{0}\epsilon_{CNM}/d_{CNM}$$

where the dielectric constant *ϵ_CNM_*=3.5^28^ and *d_CNM_*=1.2 nm. Thus the charge carrier concentration induced by the molecular field can be estimated to 3.11×10^12^ cm^−2^. From our transport measurements, we have estimated the variation in charge carrier concentration using Equation (4):

(4) $$\Delta n=\frac{C_{{SiO}_{2}}\left({\Delta V}_{Dirac\;}\right)}{e}$$

where ${C_{SiO}}_{2}$ is the capacitance of the silicon oxide layer of thickness 600 nm, which is calculated as 5.75×10^−5^ F m^−2^ and ${\Delta V}_{Dirac\;}$ is the shift in Dirac point, which is on average 35 V. The estimated change in charge carrier concentration from the transport measurement is 1.26×10^12^ cm^−2^. This result demonstrates a very fair correspondence between the estimation and the experimentally observed changes in the charge carrier concentration between due to the *trans*–*cis* conformational change in the azobenzene.

In this work, we presented a route towards optically triggered control of the charge carrier density in GFET devices by functionalizing them via the van der Waals assembly of ∼1 nm thick molecular nanosheets—azo‐CNMs. The density of azobenzene functional groups in these molecular nanosheets was obtained from XPS analysis enabling us to study the photoresponse of the devices quantitatively. By illuminating azo‐CNM/GFET devices with 365 nm light, the azobenzene molecules undergo a change from *trans* to *cis* conformation, which induces a positive voltage at the device gate resulting in a respective shift of the Dirac point towards lower voltages, that is, its effective *n*‐doping. The application of the 455 nm light induces a reversible change of the device transport characteristics. The observed modification of the charge carrier concentration in the graphene channel corresponds well with a simple model, considering the surface density of azobenzene molecules in an azo‐CNM and a respective induced electrical field due to the *trans–cis* transformation. Therewith, a new methodology towards chemical functionalization of graphene FETs with light responsive molecules is presented, which in perspective also can be adapted to other electronic two‐dimensional materials like, e.g., transition metal dichalcogenides.

## Experimental Section

### Synthesis of NH_2_‐CNMs

NH_2_‐CNMs were synthesised by electron‐beam‐induced crosslinking of 4'‐nitro‐1,1'‐biphenyl‐4‐thiol (NBPT, Taros 99 %, sublimated before use) self‐assembled monolayers (SAMs) on gold. To form the SAMs, we used 300 nm thermally evaporated Au on mica substrates (Georg Albert PVD‐Coatings). The substrates were cleaned in an oxygen plasma for 30 seconds, rinsed with ethanol (VWR, HPLC grade) and dried in a stream of nitrogen. The substrates were then immersed in a ≈0.1 mmol solution of NBPT in dry, degassed *N*,*N*‐dimethylformamide (DMF, Alfa Aeser 99.9 %) for 72 h in a sealed flask under nitrogen. After the formation of the SAM, the samples were rinsed with DMF and ethanol several times and blown dry in a nitrogen stream. Finally, the samples were irradiated using a low‐energy electron gun (FG15/40, Specs) with an energy of 100 eV and an electron dose of 50 mC cm^−2^ under high vacuum conditions (1×10^−8^ mbar). This converts the SAM into a CNM by an established electron beam induced crosslinking process.^29^

### Functionalization of NH_2_‐CNM with azobenzene molecules

Triethylamine (TEA, Sigma Aldrich 99.5 %) was used as a de‐protonating agent for amino groups. 4‐[4‐(Dimethylamino)phenylazo]benzoic acid *N*‐succinimidyl ester (azobenzene‐NHS, Sigma Aldrich 98 %) was used as an azobenzene derivative to functionalize the NH_2_‐CNMs. The NH_2_‐CNM on an Au substrate is rinsed with ethanol and then blown dry under a stream of nitrogen. Afterwards it is placed in a clean test tube along with 2 mL DMF, 10 μL TEA and ≈0.1 mg azobenzene‐NHS. The solution was then shaken for 5 minutes and put into the dark for 72 h at room temperature.

### X‐ray photoelectron spectroscopy

XPS was carried out in a Multiprobe UHV system (Scienta Omicron) using a monochromatic X‐ray source (Al K_α_, 1486.7 eV) and an electron analyser (Argus CU) with a resolution of 0.6 eV. The XP spectra were fitted using Voigt functions (30:70) after Shirley (C1s) or linear (N1s, S2p) background subtraction. The thickness of the CNMs was calculated from the attenuation of the XP Au4f signal in comparison to the Au4f signal of a clean Au reference employing the Beer–Lambert law and the attenuation length of 36 Å.^30^

### Raman spectroscopy

The Raman spectra were acquired using a Bruker Senterra spectrometer operated in backscattering mode. Measurements at 532 nm were obtained with a frequency‐doubled Nd:YAG Laser, a 50× objective and a thermoelectrically cooled CCD detector. The spectral resolution of the system is 2–3 cm^−1^. For all spectra, the Si peak at 520.7 cm^−1^ was used for peak shift calibration of the instrument.

### Fabrication steps of GFET devices

Single‐layer graphene is grown by chemical vapor deposition (CVD) on Cu foils following the protocol of Li et al.2a The fabricated graphene was delaminated from the Cu foil with the electrochemical method then transferred to highly doped silicon wafers with 600 nm dry thermal oxide. Then, Ti (2 nm)/Au (100 nm) metal contacts of two‐terminal were defined using standard e‐beam lithography and lift‐off process.

### Irradiation of GFET, NH_2_‐CNM/GFET and azo‐CNM/GFET devices with light

The light‐emitting diodes (THORLABS) with dominant wavelengths of 365 nm (M365L2) and 455 nm (M455L3) were used in this study. For irradiation of the FET devices their irradiation intensities were adjusted to ≈6 mW cm^−2^ for 365 nm LED and ≈9 mW cm^−2^ for 455 nm LED.

### Electrical characterization

The electrical characterization was carried out with Keithley 2614B/ 2634B Source Measure Units. One SMU was used to change the voltage of the gate with respect to the source/drain in the range between −80 and 150 V for the back‐gated devices in vacuum. The other SMU was used to apply the Source‐Drain Voltage of 10 mV. A lakeshore vacuum needle probe station TTPX was used to measure the devices in vacuum with back gate.

## Conflict of interest

The authors declare no conflict of interest.

## Supporting information

As a service to our authors and readers, this journal provides supporting information supplied by the authors. Such materials are peer reviewed and may be re‐organized for online delivery, but are not copy‐edited or typeset. Technical support issues arising from supporting information (other than missing files) should be addressed to the authors.

Supplementary
