# Supplementary material for: Optically Triggered Control of the Charge Carrier Density in Chemically Functionalized Graphene Field Effect Transistors
Source: Chemistry. 2020 Mar 27;26(29):6473–8. doi: 10.1002/chem.202000431 (PMC7318135; doi:10.1002/chem.202000431)
Supplement: Supplementary file 1 — Supplementary [file CHEM-26-6473-s001.pdf]

Chemistry–A European Journal

Supporting Information

Optically Triggered Control of the Charge Carrier Density in Chemically Functionalized Graphene Field Effect Transistors

Zian Tang,[a] Antony George,[a] Andreas Winter,[a] David Kaiser,[a] Christof Neumann,[a] Thomas Weimann,[b] and Andrey Turchanin*[a, c]

Supporting Information (SI)

Optically triggered control of the charge carrier density in chemically functionalized graphene field effect transistors

Zian Tang[1], Antony George[1], Andreas Winter[1], David Kaiser[1], Christof Neumann[1], Thomas Weimann[2], Andrey Turchanin[1,3]

[1]*Institute of Physical Chemistry, Friedrich Schiller University Jena,*

Lessingstr. 10, 07743 Jena, Germany

[2]*Physikalisch-Technische Bundesanstalt (PTB), Bundesallee 100, 38116 Braunschweig, Germany*

[3]*Jena Center for Soft Matter, Philosophenweg 7, 07743 Jena, Germany*

Corresponding author:

Prof. Dr. Andrey Turchanin

Email: andrey.turchanin@uni-jena.de

Estimation of the functionalization efficiency of NH_2-CNMs with azobenzene

Thickness analysis and calculation of functionalization efficiently are based on the Beer-Lambert law for XPS [1]

$$I_s = I_0 \exp(-d/\lambda\cos\theta), \qquad (1)$$

where θ value is 18.7°, the angle between the sample normal and the detector. For the N 1s signal in aromatic carbon, we use the attenuation length λ = 2.1 nm. [2] The functionalization efficiency was calculated based on two assumptions:

1) As shown in Scheme S1a, we assume that the immobilized *trans*-azobenzene molecules are grafted homogeneously on a NH_2-CNM. Then, the N 1s signal from all nitrogen on NH_2-CNM is attenuated by the immobilized *trans*-azobenzene layer (thickness: 0.2 nm) homogeneously and the N 1s signal from azo nitrogen is only attenuated by half of the azobenzene layer (thickness 0.1 nm). Based on Equation 1 we obtain:

$$\frac{I_{0.2}}{I_0} = \exp\left(-\frac{0.2}{2.1\cos 1\ \ .7°}\right) = 0.90,$$

$$\frac{I_{0.1}}{I_0} = \exp\left(-\frac{0.1}{2.1\cos 18.7°}\right) = 0.95.$$

We set the percentage of functionalized amino groups as x and the percentage of non-functionalized amino groups as y, then

$$x + y = 100\%.$$

According to the XPS data presented in Figure 2a, the ratio of amide + azo nitrogen to tertiary amine + amino nitrogen is 1:2, thus

$$\frac{0.90x + 2\times 0.95x}{x + 0.9y} = \frac{1}{2},$$

and

$$x \approx 16\%.$$

2) As shown in Scheme S1b, one can assume that the grafted *trans*-azobenzene molecules cover only some area of a NH_2-CNM. In this case, they attenuate only the N 1s signal from amide nitrogen and one of the amino nitrogen next to it, and the azo nitrogen (orange) signal is only attenuated by a half of the azobenzene molecule (the length of the azobenzene molecule is 1.3 nm). Based on Equation 1 we obtain:

$$\frac{I_{1.3}}{I_0} = \exp\left(-\frac{1.3}{2.1\cos 18.7°}\right) = 0.52$$

$$\frac{I_{0.7}}{I_0} = \exp\left(-\frac{0.7}{2.1\cos 18.7°}\right) = 0.70$$

We set the percentage of the functionalized amino groups to x and the percentage of the non-functionalized amino groups to y, then

$$x + y = 100\%.$$

Using a similar calculation method as in 1), we obtain:

$$\frac{0.52x+2\times0.70x}{x+0.52x+(y-x)} = \frac{1}{2},$$

and

$$x \approx 23\%$$

Therewith the percentage of the amino groups in a NH_2-CNM is functionalized with azobenzene molecules is estimated to be 20±3%.

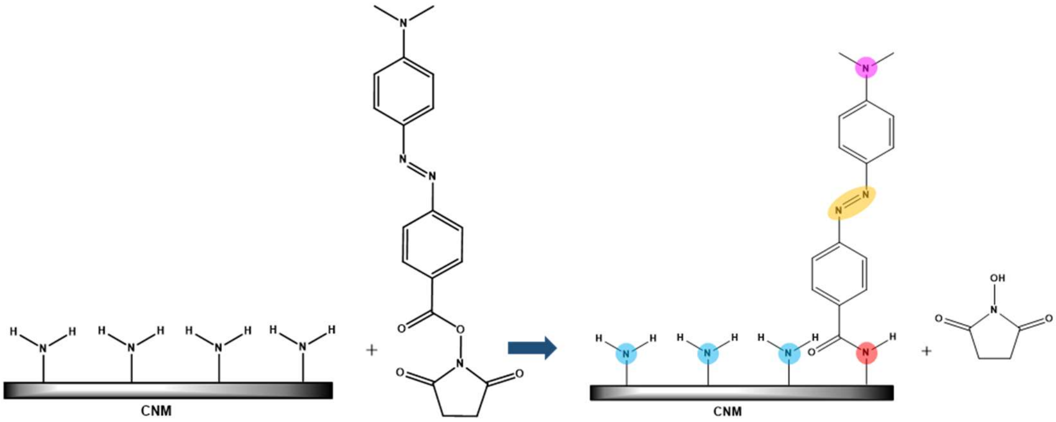

Figure S1. Schematic representation of the NHS ester coupling reaction for the attachment of azobenzene molecules to a carbon nanomembrane (NH_2-CNM). The red nitrogen atom is the formed amide group; the blue nitrogen atoms are the remaining non-functionalized amino groups; the azo nitrogen is yellow and the tertiary amine nitrogen is purple.

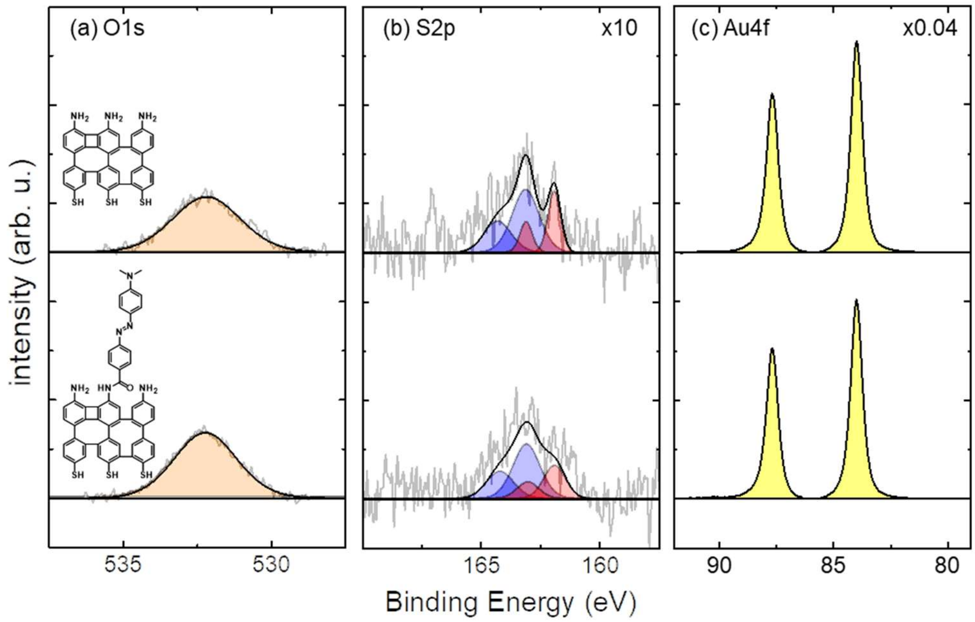

Figure S2. High-resolution XP spectra of NH_2-CNM and azo-CNM: a) O1s, b) S2p and c) Au 4f. The peak at 162.0 eV is due to the thiolate sulfur, while the peak at 163.1 eV is assigned to the free thiol and disulfide species.[3]

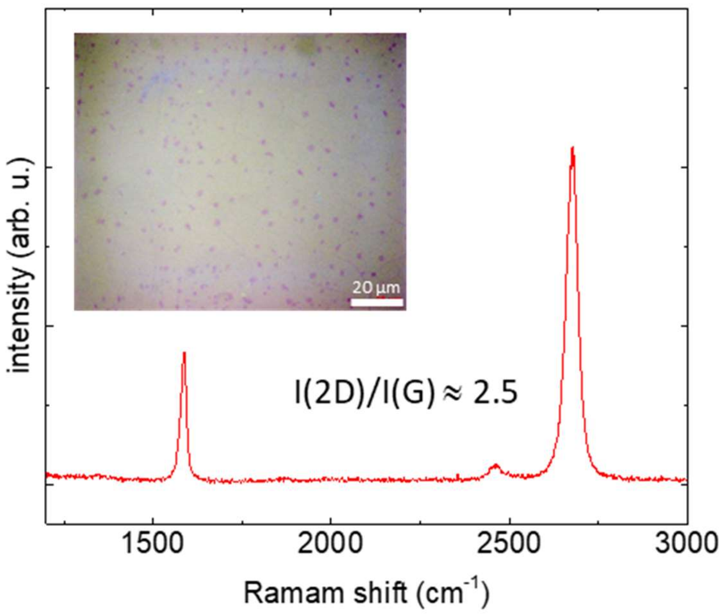

Figure S3. Raman spectrum (λ = 532 nm) and optical microscope image (insert) of a CVD grown single layer graphene sample transferred onto a SiO_2/Si wafer used for the fabrication of GFETs.

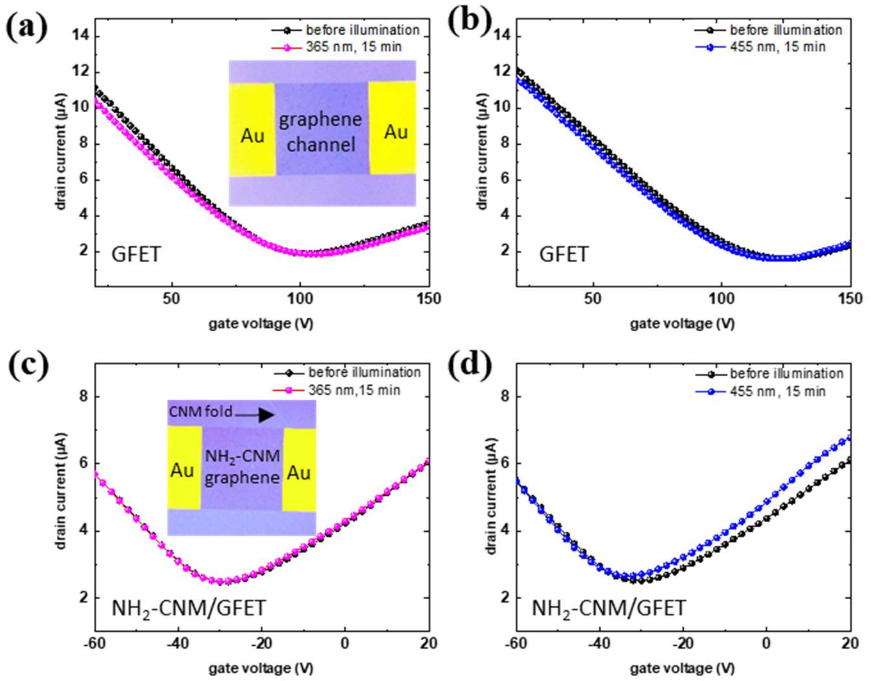

Figure S4: Control experiments performed with GFET and NH_2-CNM/GFET devices to confirm that their illumination with 365 nm and 455 nm light does not induce any recognizable changed in the transfer curves; (a-b) GFET; (c-d) NH_2-CNM/GFET. Inserts in (a) and (b) are the optical microscope images of typical GFET and NH_2-CNM/GFET devices, respectively, used in this study with gold source and drain contacts.

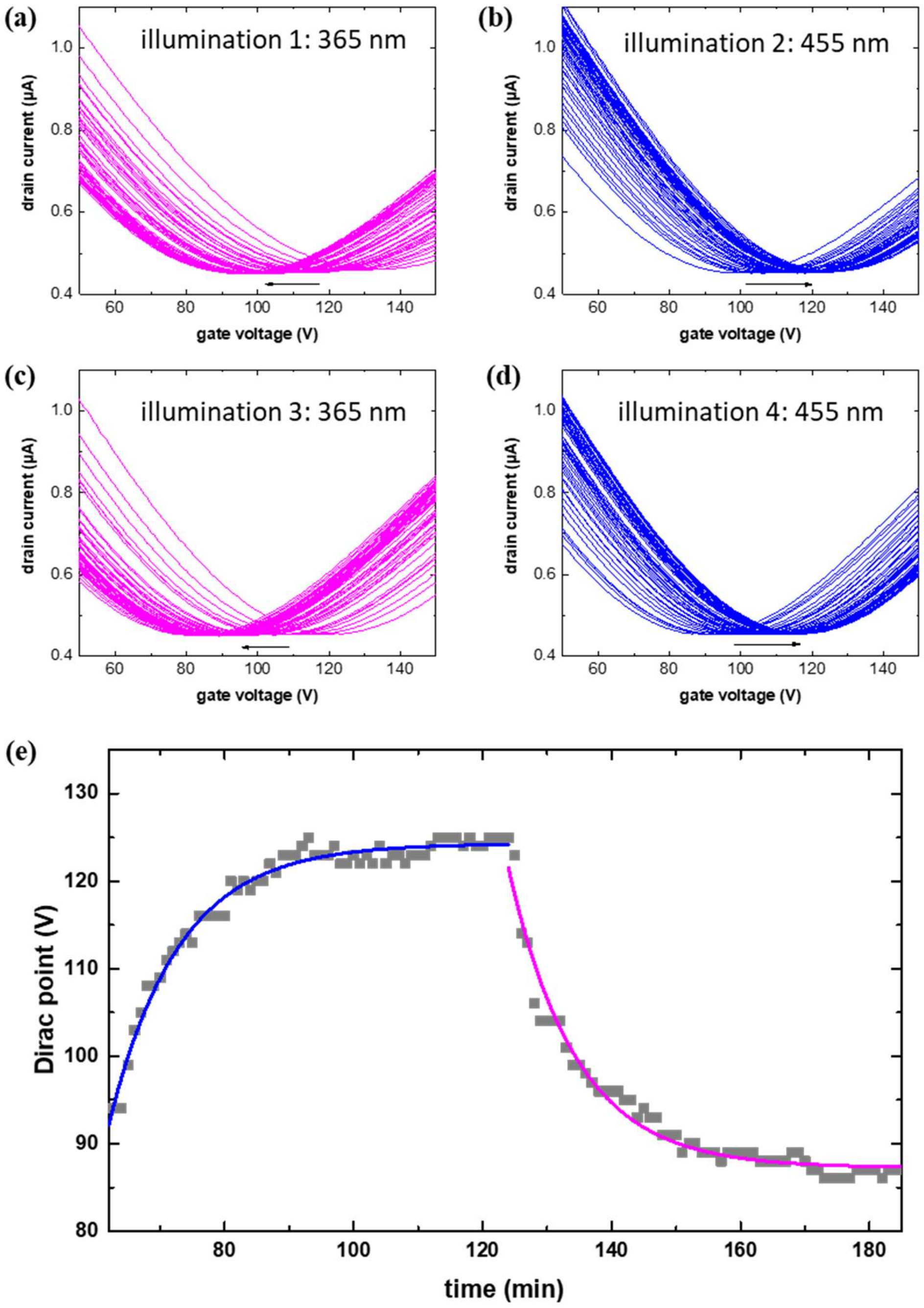

Figure S5: (a-d) Transfer curves of the azo-CNM/GFET device presented in Figure 3b of the main manuscript. (e) Time dependent shift of Dirac point is fitted with single exponential functions (see main manuscript for more details).

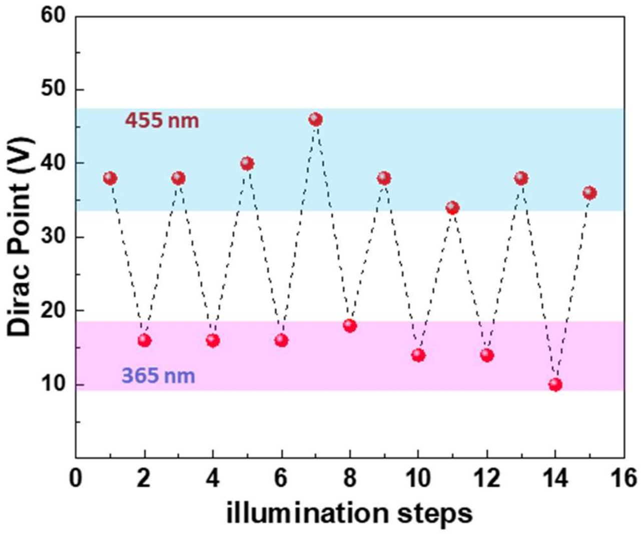

Figure S6: Change of the Dirac point of an azo-CNM/GFET device upon sequential exposures with 365 nm and 455 nm light. The duration of each exposure was 15 min. The transfer characteristics were recorded afterwards immediately under dark condition.

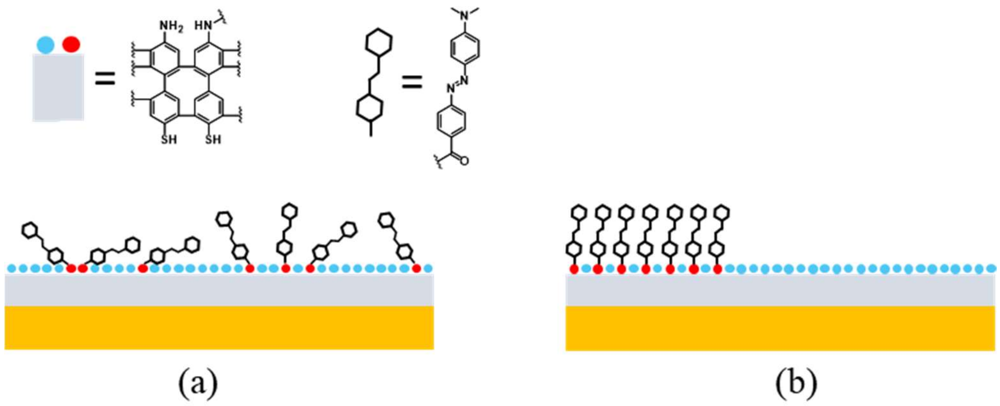

Scheme S1: Schematic representation of two hypothetical scenarios of the azobenzene grafting on a NH_2-CNM: (a) homogenous grafting; (b) formation if islands.

Table S1: Quantitative analysis of the high-resolution XP spectra of the NH_2-CNM and azo-CNM samples on Au presented in Figure 2 and Figure S2 including the peak assignment, their binding energies, full width at half maximum (FWHM) values and areas obtained from the spectra deconvolution. The peak fitting of the sulfur doublet was performed using the fixed intensity ratios due to the spin-orbit coupling of the p-photoelectrons. For the elemental ratios mentioned in the main paper the relative sensitivity factors (RSF) of 1 for C 1s, 1.8 for N 1s, 2.9 for O 1s and 1.11 for S $2p_{3/2}$ were used.

Peak assignment	Binding energy, eV	FWHM, eV	Area, %
**S $2p_{3/2}$**			
**CNM**			
thiolate	161.9	0.7	33
disulfide	163.1	1.3	67
**azo-CNM**			
thiolate	161.9	1.1	33
disulfide	163.1	1.4	67
**C 1s**			
**CNM**			
C-C aromatic	284.3	1.3	73
C-S/C-N/C-C aliphatic	285.5	1.8	18
C=O	286.9	1.8	5
O-C=O	288.3	2.0	4
**azo-CNM**			
C-C aromatic	284.3	1.3	65
C-S/C-N/C-C aliphatic	285.5	1.8	22
C=O	286.9	1.7	5
O-C=O	288.1	2.5	8
**N 1s**			
**CNM**			
$-NH_2$	399.4	2.3	100
**azo-CNM**			
-NH2/Tertiary amine	399.1	1.7	67

C-N=N-C/Amide	400.4	1.2	33
**O 1s**			
**CNM**			
C=O/ O-C=O/C-O-C/C-OH	532.2	2.7	100
**azo-CNM**			
C=O/ O-C=O/C-O-C/C-OH	532.2	2.5	100
**Au $4f_{7/2}$**			
**CNM**			
Au	84.0	Total area: 13979 a.u.	
**azo-CNM**			
Au	84.0	Total area: 13215 a.u.	
**bare gold**			
Au	84.0	Total area: 20050 a.u.	

References

[1] J. W. John F. Watts in *An Introduction to Surface Analysis by XPS and AES*, John Wiley & Sons, Ltd, **2003**, pp. 79-111.

[2] V. Stadler, Doctoral dissertation, Chemische Nanolithographie mit Elektronenstrahlen an Biphenyl Monoschichten (eng.: Chemical nanolithography with electron beams on biphenyl monolayers), Ruprecht Karls University Heidelberg, Heidelberg, **2001**, p. 131.

[3] A. Turchanin, D. Käfer, M. El-Desawy, C. Wöll, G. Witte and A. Gölzhäuser, *Langmuir* **2009**, *25*, 7342-7352.
